# Does the crisis intervention team (CIT) training improve police officers’ knowledge, attitude, and mental health stigma?

**DOI:** 10.1192/j.eurpsy.2021.1246

**Published:** 2021-08-13

**Authors:** N. Veluri, Z. Mansuri

**Affiliations:** 1 N/a, American University of Integrative Sciences, School of Medicine, St.Michale, Barbados; 2 Department Of Psychiatry, Boston Children’s Hospital/Harvard Medical School, Boston, United States of America

**Keywords:** crisis intervention team training, crisis intervention team, mental health education, mental health stigma

## Abstract

**Introduction:**

The Crisis Intervention Team (CIT) training was developed to educate police officers regarding the complexity of mental health (MHI) issues, and better prepare them for crisis encounters with persons with mental illness (PwMI).

**Objectives:**

To determine if CIT training improves police officers’ knowledge, attitude, and stigma about mental health issues.

**Methods:**

A systematic review followed the PRISMA protocol and was conducted on the PubMed database (Figure 1). Search strings were “crisis intervention team training,” “crisis intervention team,” “CIT,” “effectiveness,” and “police.” Inclusion eligibility required primary studies using surveys that measured the CIT training outcomes (i.e., knowledge, attitude, and stigma). Literature/narrative reviews or opinions were excluded.

**Figure:**
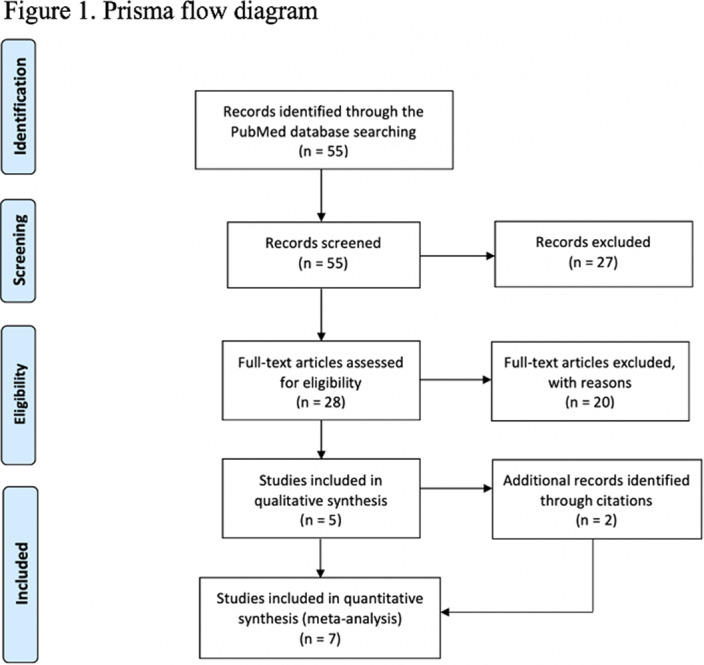

**Results:**

The comprehensive meta-analysis software version 3.0 was utilized during analysis. Randomized odds ratios using a 95% confidence interval (CI) were obtained. Officers’ scores for knowledge, attitude, and stigma about MHI were taken before and after the survey. The Control group consisted of officers without CIT training. The CIT trained officers displayed an improvement in knowledge (OR 2.35, CI: 1.51– 3.67), attitude (OR 2.55, CI: 1.36–4.78), and stigma (OR 3.11, CI: 1.88–5.17). The results were statistically significant, with a p-value of less than 0.001 (Figure 2).

**Figure:**
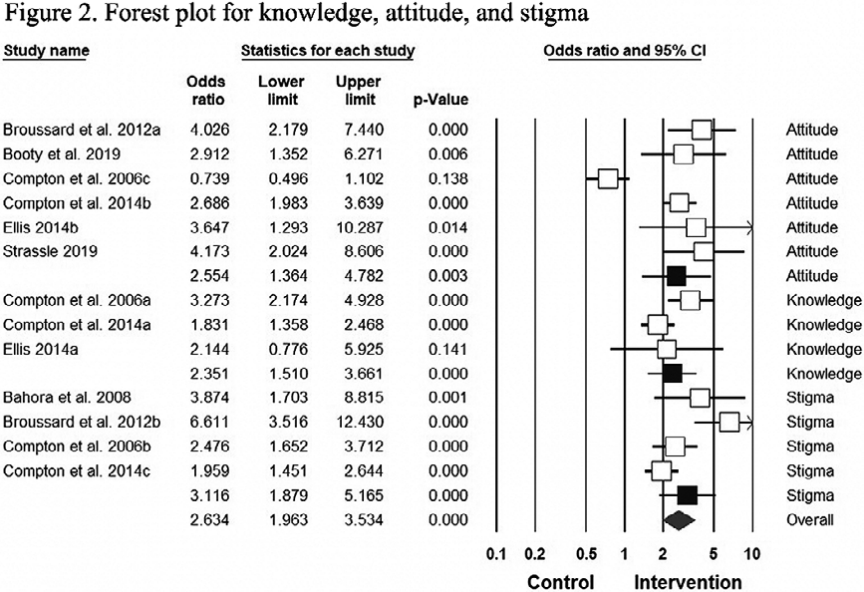

**Conclusions:**

CIT trained police officers displayed a significant improvement in their knowledge, attitude, and reduced stigma towards PwMI. Although our study displays CIT training’s positive effects, previous studies reported a nullified effect of CIT in reducing arrests and the use of force during police officers encounters with PwMI. Future researchers must explore this gap, mainly focusing on gender and race bias.

